# Preparation methodology evaluation of rat pulmonary tissues containing mineral fibers following inhalation exposure to Libby amphibole asbestos

**DOI:** 10.1186/s12995-025-00476-3

**Published:** 2025-10-14

**Authors:** Jamie S. Richey, John R. Shaw, Amit Gupta, Dawn M. Fallacara, Barney R. Sparrow, Anbo Wang, Karen E. Elsass, Georgia K. Roberts, Pei-Li Yao, Matthew D. Stout, Benjamin J. Ellis, Robyn L. Ray

**Affiliations:** 1 Battelle Memorial Institute, Columbus, OH USA; 2 AmplifyBio, West Jefferson, OH USA; 3 Division of Translational Toxicology, National Institute of Environmental Health Sciences, Research Triangle Park, NC USA; 4 Ramboll, Princeton, NJ USA; 5 EMSL Analytical Inc, Cinnaminson, NJ USA

**Keywords:** Fiber burden, Inhaled mineral fiber, Chemical digestion, High temperature ashing, Asbestos, Libby amphibole

## Abstract

**Background:**

Inhaled mineral fibers including asbestos are associated with lung cancer and pleural disease. In this study, we evaluated methodologies for mineral fiber isolation with subsequent physical and chemical characterization from pulmonary tissues of rats exposed to Libby amphibole asbestos 2007 (LA 2007) fibers via repeated nose-only inhalation. At the completion of the exposures, lungs were collected either as is or instilled with liquid agarose to produce a pulmonary cast. To extract fibers, lung tissue with and without pulmonary casts were further processed by either high temperature ashing or chemical digestion. The use of liquid agarose to produce pulmonary casts was discontinued after the first study assessment as no fibers were present in the pleural cavity for evaluation. Fibers isolated from the lungs were analyzed by transmission electron microscopy (TEM) coupled with selected area electron diffraction (SAED) and energy dispersive X-ray spectroscopy (EDS) for physical and chemical characterization. The bulk LA 2007 test material was also analyzed to provide comparison of fiber dimensions and chemical composition of the fibers introduced during exposure. Particular interest was focused on the comparison between high temperature ashing and chemical digestion extraction methodologies.

**Results:**

Chemical digestion of lung tissue with and without pulmonary casts resulted in fiber dimensions and chemical profiles similar to the bulk LA 2007 test chemical and exposure atmosphere. Conversely, high temperature ashing resulted in degraded fibers with chemically altered profiles.

**Conclusions:**

Based on the findings in this study, chemical digestion of lung tissue is the preferred preparation method for the isolation of inhaled mineral fibers for lung burden analysis.

## Background

Human studies have shown that asbestos exposure is associated with a spectrum of pulmonary lesions including lung cancer, mesothelioma, and pleural disease [16, 17, 21]. Although the hazards of asbestos exposure to humans are well recognized, the lack of comprehensive and high-quality rodent toxicology data on asbestos reduces the opportunity to make regulatory determinations and facilitate risk management. Notably, there is a need to understand how chemical and physical differences across fibers impact the dose-responses of toxicologically relevant endpoints; the National Institute of Environmental Health Sciences (NIEHS) has initiated a research effort to address this gap.

The NIEHS has conducted a series of inhalation studies to assess the adverse health effects of Libby amphibole asbestos 2007 (referred to as LA 2007 throughout this paper), a test material representing the air from the vermiculite mine near Libby, MT [13]. The overall objectives for these studies were to (1) generate in vivo data to complement robust chemical/physical characterization and human health effect outcomes on LA 2007, and (2) address fundamental questions around exposure-response to advance the understanding of fiber toxicology and risk assessment in general. The development of a nose-only inhalation exposure system consisting of a single generator for assessing health effects of LA 2007 on rodents with detailed examination on aerosolized asbestos fibers in the exposure atmosphere and bulk material was previously reported [24, 25] Part B, [13]. To accomplish the goals of the study, it was critical to generate accurate data for fiber count, size measurements, and chemistry/mineralogy and to compare these to the exposure atmosphere and bulk material, as well as to evaluate the kinetics of inhaled material in the lung. To achieve this goal, methodologies unique to the isolation and preservation of fibers from the biological matrix without altering the original inhaled structures of the material were needed.

Physical and chemical properties of individual mineral fibers isolated from tissues can be assessed by transmission electron microscopy (TEM) coupled with selected area electron diffraction (SAED) and energy dispersive x-ray spectroscopy (EDS). This approach has been well-recognized for fiber characterization [22] and was used to characterize fibers in the bulk and exposure atmosphere [24, 25] Part B). TEM is known to have a higher optimal spatial resolution for observing inner structure and ideal for detecting thinner fibers. Therefore, TEM is the preferred method to evaluate smaller fibers in terms of understanding their crystal morphology compared to other less sensitive microscopic methods, such as scanning electron microscope (SEM), for detecting the surface. To date, tissue preparation methodology for TEM analysis of asbestos fibers commonly involved either a dry ashing process at low or high temperatures [7, 10, 11] or chemical digestion with potassium hydroxide, sodium hypochlorite or formamide [9, 10, 19, 26, 27] to efficiently remove the biological matrix while minimizing the damage to asbestos fibers. In some cases, the combination of heating and chemical treatment was applied [3, 26]. Both techniques are widely accepted for fiber extraction, although no conclusive determination was made to directly compare the suitability of these two distinct approaches on fiber characterization. In general, the high-temperature ashing technique is faster and requires less intensive effort. However, ashing methods may not be satisfactory as a prolonged ashing duration can cause artefactual fragmentation of fibers [18]. One study has shown that hot-temperature ashing for a short period of time resulted in a slightly higher fiber count compared to wet chemical digestion for 2–3 days; however, the difference did not reach statistical significance [10]. It is also worth noting that excess procedures for sample preparation may introduce unnecessary modifications to fiber counts, chemistry and/or size distribution [4].

Preliminary evaluations were performed prior to the studies discussed herein using dry ashing and chemical digestion methods on fiber free lung tissue or lung tissue spiked with asbestos fibers. From these evaluations, the low-temperature ashing method was not considered viable primarily due to time constraints when compared to high-temperature ashing and chemical digestion methods (data not shown). In fact, the lengthy procedure for low-temperature ashing negatively affected the ability to obtain a sufficient quantity of sample sets for analysis.

In the current study, the evaluation of various methodologies for isolating inhaled mineral fibers from pulmonary tissues in rats exposed to LA 2007 via nose-only inhalation is presented. The results of physical size (length, width, aspect ratio) and chemical composition determination for recovered inhaled asbestos fibers were compared to the pre-aerosolized mineral fibers (bulk material). The primary goals for this study were (1) to determine appropriate techniques to isolate asbestos fibers from biological specimens while maintaining fibers as close to their natural state as possible for subsequent toxicological studies; and (2) to elucidate the potential of artefact generation due to isolation methodologies. The present study provides comprehensive comparisons between high-temperature ashing (referred to throughout as ashing) and chemical digestion methods for fiber recovery from exposed rodent lungs with and without agarose gel casting where lungs were infused with agarose gels to create solid casts, a common approach to recover fibers from the pleural cavity [2, 6]. It is also anticipated that the results from this study may benefit the evaluation of other natural mineral fibers of concern present in the environment.

## Methods

### Test Materials

Libby amphibole asbestos 2007 (referred to as LA 2007) was provided by the National Institute of Environmental Health Sciences (NIEHS) via the United States Environmental Protection Agency (EPA, Saint Louis, MO). The initial characterization of the LA 2007 material was performed by Meeker [14] and Lowers (USGS, [13]). Subsequent characterization performed by Battelle and EMSL Analytical, Inc [25] indicated the bulk LA 2007 material was composed of mineral fibers with an average length of 4.71 μm, average width of 0.27 μm, and was crystalline with electron diffraction patterns consistent with amphibole asbestos collected from Libby, MT (known as LA) in 2007. The elemental oxide and mineral composition of the bulk material was consistent with the characterization of LA 2007 presented by the USGS and Meeker and primarily contained winchite, richterite, and tremolite [25] . The analysis reported by Wang et al. [24, 25] showed the fibers present in the exposure atmosphere were similar to the fibers present in the bulk material.

### Animals, Exposure, and Preparation of Lungs

Two inhalation exposure feasibility studies were conducted to expose rats to LA 2007 via repeated nose-only inhalation at target concentrations of 0, 0.1 or 10 mg/m^3^ followed by tissue collection and evaluation. The primary goal of these studies were to determine the feasibility and tolerability of inhalation exposure of LA 2007 and other asbestos-like materials to rodents for subsequent toxicity and carcinogenicity studies. The toxicology studies for LA 2007 were designed with a tiered approach to occur in multiple phases, including prestudy characterization work, to iteratively evaluate design and results and to allow modifications of the exposure system as it was being built and tested. This resulted in a time lag between the two exposure studies as the study design of the toxicology studies evolved and the lessons learned from the first study were applied to the second study. One example of this step-wise approach was the development of the inhalation exposure system. Exposure Study 1 utilized a prototype single-carousel exposure system for rat feasibility and tolerability evaluations/confirmation prior to moving forward with efforts to build the larger six-carousel exposure system to be used for toxicology studies (as utilized in Exposure Study 2). In addition, the primary focus of these studies as presented in this manuscript was to determine a suitable digestion approach to retrieve fibers from biological tissues for fiber characterization, which is a key element of the subsequent toxicological studies. The *de minimus* rule for the number of rats utilized was applied for this preliminary testing as the primary focus of the preparation method evaluations was the gross observation of the quality of fibers present in the lungs following different digestion methods. In this regard, the statistical analysis performed is based on the number of fibers evaluated and not the number of lungs utilized. With this, the small number of animals/lung lobes utilized for analysis was sufficient for this purpose. This approach allowed for the utilization of tissues collected from animals exposed to LA 2007 fibers without excess exposures being conducted and prevented potential fiber overload in the lungs, which would have had a negative impact on the TEM analyses. The studies were reviewed and approved by Battelle’s Institutional Animal Care and Use Committee (IACUC) prior to conduct.

#### Exposure Study 1

The first exposure study was conducted utilizing a prototype nose-only inhalation exposure system consisting of a single exposure carousel with a single aerosol generator designed to deliver LA 2007 aerosol to rats at a target aerosol concentration of 10 mg/m^3^ [24]. An additional nose-only inhalation exposure system was designed to expose rats to HEPA filtered air (0 mg/m^3^) as a control group. Each exposure group was comprised of three male Wistar Han rats exposed to LA 2007 at time weighted target aerosol concentrations of either 0 (for 180 min), 0.1 (for 2 min), or 10 (for 180 min) mg/m^3^ for 5 consecutive days. At the completion of the exposure period, the animals were humanely terminated and the lungs from each animal were infused with warm liquid agarose solution (2.5 mL) to inflate the lungs. The agarose inflated lungs were allowed to cure by placing the animal carcass on ice for approximately 30 min. Afterward, the lungs were removed and the lungs (three left and three right lobes for each exposure concentration, three concentrations) was stored frozen (approximately − 70 °C) prior to submission for fiber isolation and burden determination using either high temperature ashing or chemical digestion as the preparation technique.

#### Exposure Study 2

Following evaluation of the observations from the first study, a second exposure study was conducted utilizing the full-scale nose-only inhalation exposure system consisting of five exposure carousels with a single aerosol generator designed to deliver LA 2007 aerosol to rats over a concentration range of 0.1 to 10 mg/m^3^ [25]. An additional nose-only inhalation carousel was designed to expose rats to HEPA filtered air only (0 mg/m^3^) as a control group. Each exposure group was comprised of two male and two female Sprague Dawley (Hsd: Sprague Dawley SD) rats exposed to either 0 or 10 mg/m^3^ LA 2007 for 180 min each day for 3 consecutive days. At the completion of the exposure period, the animals were humanely terminated and the lungs from each animal were removed without infusion of agarose. The pleural casting process from the first study was discontinued due to (1) the absence of fibers present in the pleural cavity from Exposure Study 1 and (2) the observed potential interference/fiber interactions with the agarose gel in the lungs as described below. The lungs (four left and four right lobes for each exposure concentration, two concentrations) was stored frozen (approximately − 70 °C) prior to submission for fiber isolation and burden determination using either high temperature ashing or chemical digestion as the preparation technique.

Changes to the study design between these two exposure studies, such as the rat strain and sexes utilized, reflected the evolution in the design of subsequent toxicology studies on LA 2007 over time. Based on the observations from Study 1, the implementation of the shortened exposure period was found to be appropriate along with the exclusion of the 0.1 mg/m^3^ dose group. Excluding the low concentration dose group reduced the number of animals needed once assurance was observed that a sufficient number of fibers were retained in the lungs for the digestion process evaluation. Shortening the exposure duration from 5 days to 3 days further eliminated excess exposures being conducted and prevented potential fiber overload in the lungs, which would have had a negative impact on the TEM analyses.

A summary of the exposure studies conducted is shown in Table 1.

**Table 1:** Summary of Libby amphibole asbestos 2007 inhalation exposure studies

Exposure Study	Exposure Design	Animal/Age at Exposure	Tissue Collection	Preparation Methods
1	Time weighted exposure at approximately 0, 0.1, or 10 mg/m^3^ LA 2007 for 5 consecutive days • 0.1 mg/m^3^ − 2 min per day • 0 and 10 mg/m^3^ − 180 min per day	Male Wistar Han rats, 6 weeks at exposure^a^	Lung with pulmonary cast • Left and right lobes randomized per preparation method	• High Temperature Ashing (9 lobes, 3 for each concentration) • Chemical Digestion (9 lobes, 3 for each concentration)
2	Exposure at approximately 0 or 10 mg/m^3^ LA 2007 for 180 min per day for 3 consecutive days	Male and female Sprague Dawley rats, 3–4 weeks at exposure^a^	Lung without pulmonary cast • Left and right lobes randomized per preparation method	• High Temperature Ashing (8 lobes, 4 for each concentration) • Chemical Digestion (8 lobes, 4 for each concentration)

^a^Rats obtained from Envigo, Indianapolis, IN

### Mineral fiber isolation and physical and chemical characterization

Lung samples collected from exposed animals were shipped frozen on dry ice to EMSL Analytical Inc. (Cinnaminson, NJ) for fiber isolation and analysis via TEM with SAED and EDS. The lungs were divided into left and right lobes and the lobes randomized for fiber isolation using either high temperature ashing or chemical digestion. Due to the limited number of exposed lobes available for analysis and the nature of the testing performed (complete sample dissolution), not all conditions were tested with the same lobes or with the same number of lobes. For Study 1, the number of lobes alternated between *n* = 1 or 2 for left and right lobes used for each preparation technique at each exposure concentration. For Study 2, the number of lobes available was the same for both sample preparation methods (*n* = 8 per concentration) and were randomized so that each preparation technique tested included one male and one female left and right lobe, respectively, per concentration.

For high temperature ashing, each lung lobe was placed into a porcelain crucible within a cold muffle furnace. The temperature within the furnace was slowly raised over approximately 5h to a target temperature of 485°C and held at constant temperature for approximately 6h until the samples were fully reduced to ash. The furnace was allowed to cool to ambient temperature and the crucibles removed. The ash was treated with 1N hydrochloric acid and the residue brought to volume in deionized fiber-free water followed by filtration through a 0.4-µm mixed cellulose ester (MCE) membrane filter.

For chemical digestion, each lung lobe was dissected into approximately 1 cm³ cubed pieces and placed into a lidded particle free container. Sodium hypochlorite (9.2%) was added to the sample in a ratio with an excess of sodium hypochlorite present. The sample was agitated until the sample was visually digested. The digestate was filtered through a 0.4-µm polycarbonate (PC) filter followed by a rinse with ethanol. The filter was serially washed with deionized fiber-free water, ethanol/potassium permanganate solution, and aqueous oxalic acid. The PC filter was then rinsed and sonicated in deionized fiber-free water and the rinse solution filtered through a 0.4-µm MCE filter. The transfer from the PC filter to the MCE filter was necessary due to the entire lobe of lung being digested and there were too many fibers to count and size from a direct preparation of the PC digestion filter.

The slide preparation of the filters for microscopy was the same for both isolation methods. After fiber isolation, wedges of the dried MCE filter were affixed onto glass microscope slides, collapsed by hot acetone vapor, plasma-etched in an oxygen plasma, and carbon coated. The carbon coated sample was positioned onto a copper TEM grid and placed into an acetone Jaffe Wick washer where the filter membrane was dissolved. The remaining carbon film on the grid was analyzed by TEM.

An aliquot of the bulk test material used for exposure was also analyzed as reported by Wang et al. [24, 25] to provide a baseline comparison for fiber dimension and chemical composition of fibers observed in the lungs.

The primary analytical techniques for asbestos mineral fiber counting and analysis were consistent with the analytical techniques used for analysis of pre- and post-aerosolized/pre-inhaled samples reported by Wang et al. [8, 24, 25] to document the length, width, and structure type. For Study 1, a minimum of 50 structures per lobe were counted. For Study 2, the number of counted structures was increased to a minimum of 300 structures per lobe to improve statistical significance. Once a structure was encountered, it was classified as a compact cluster/agglomerate or a fiber and the length and width of the structure was recorded. All fibers ≥ 0.5 μm in length with an aspect ratio of at least 3:1 were counted. Fibers were classified as phase contrast microscopy equivalent (PCMe) as defined in the ISO 10312 method as a fiber or bundle greater than 5 μm in length and a width between 0.2 and 3.0 μm. Representative TEM images of fiber morphology, SAED images, and EDS spectra were collected for each type of fiber identified. For Study 1, one representative TEM image and EDS spectrum was collected for each lobe. For Study 2, 20 representative TEM and SAED images and EDS spectra was collected for each lobe to improve statistical significance. The TEM/EDS system calibrations are checked using several industry-standard approaches involving NIST traceable standards to confirm elemental peak alignment, peak resolution, magnification settings, and detector response sensitivity. The timing of these various calibration checks range from weekly to quarterly and any system component recalibration is performed according to EMSL’s documentation requirements. All EDS spectra of materials prepared on the TEM grids contained Cu peaks (due to grid background) at ca. 8 and 9 keV. These peaks were not included in the determination of elemental composition by EDS.

## Results

### High temperature ashing vs. Chemical digestion of lung lobe samples

**Fig. 1:**
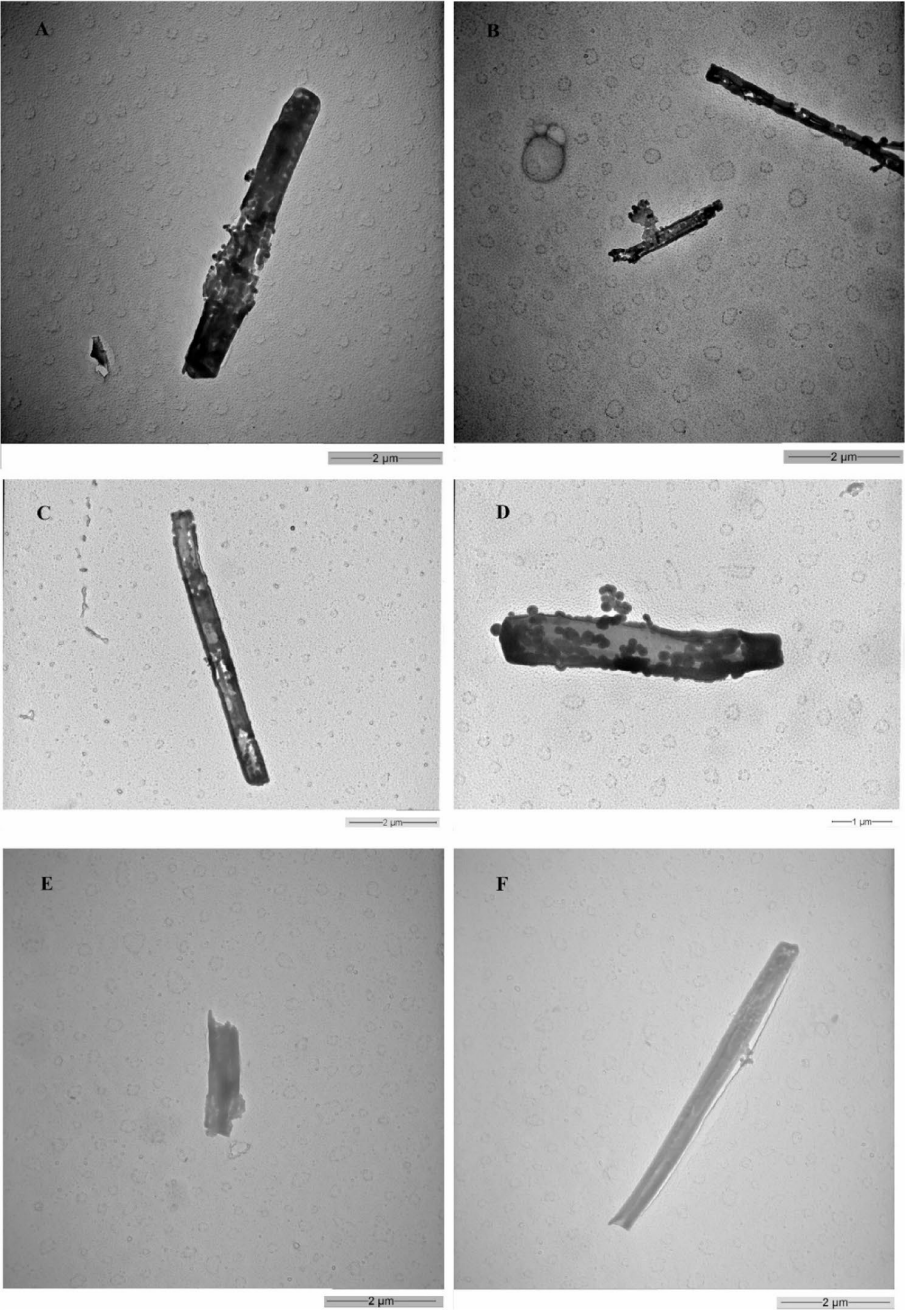
TEM Images of Ashed Lung Fibers Showing NAM Fibers (**A**-**D**) and LA Fibers (**E**-**F**)

**Fig. 2:**
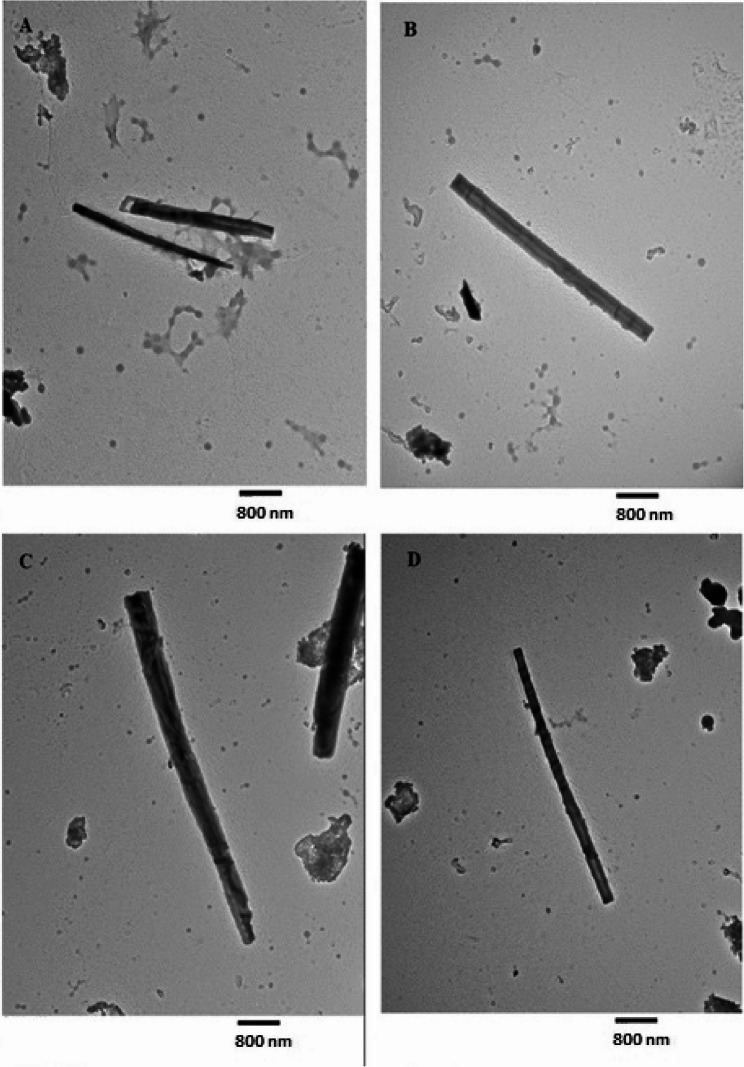
TEM Images of Fibers from Chemical Digested Lungs Classified as LA (A-D)

**Fig. 3:**
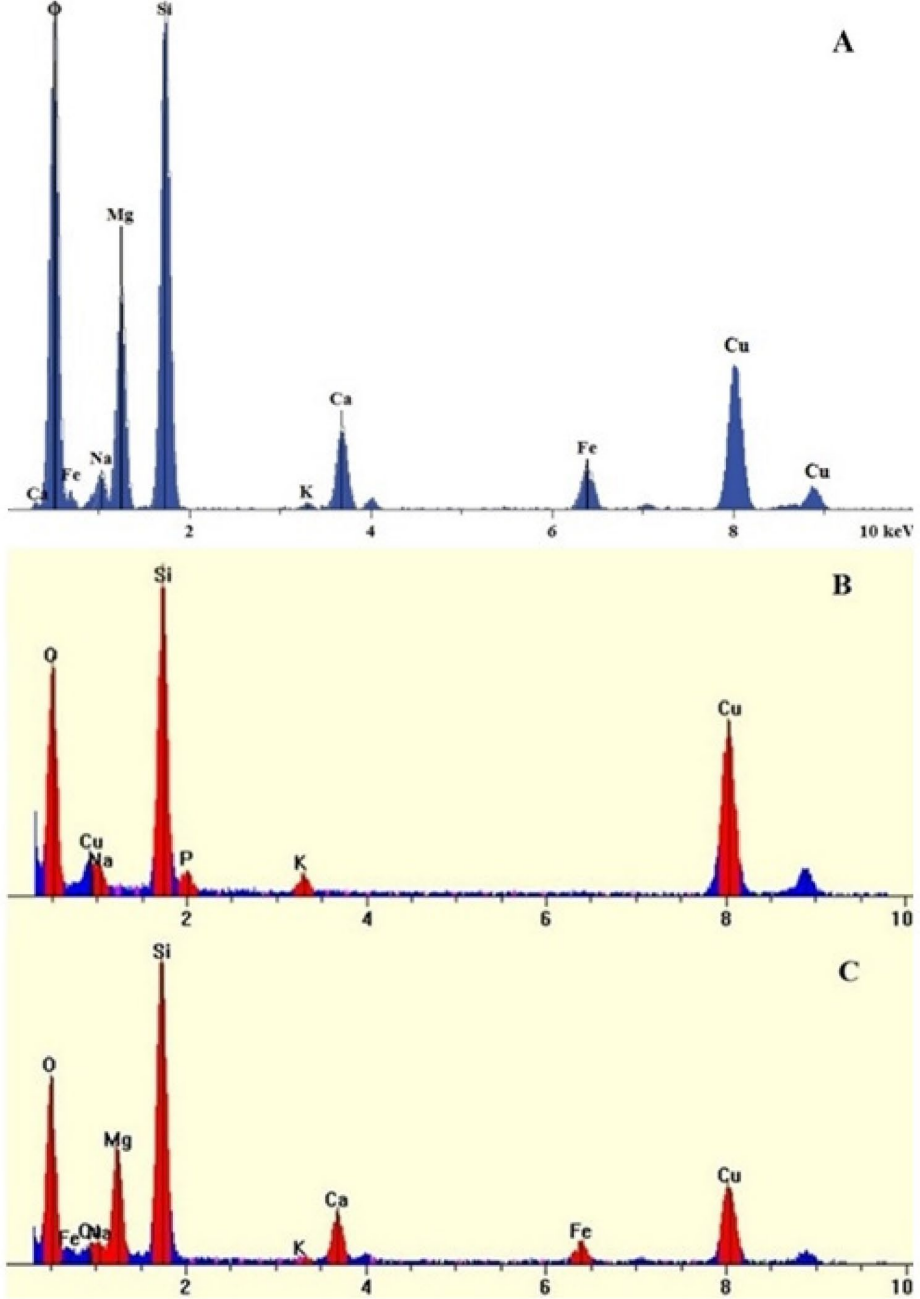
EDS Spectra (**A**) Pre-aerosolized LA, (**B**) Ashed Lung NAM Fibers, (**C**) Digested Lung LA Fibers

During the analysis of fibers from the exposed lung lobe samples, a notable and consistent difference in the appearance and composition of the mineral fibers was apparent between the LA 2007 exposed lung lobes prepared by high temperature ashing (ashed) relative to samples subjected to chemical digestion for both studies. It is worth noting that there were no obvious differences in the chemical/physical properties using a given processing method between the two studies and as such representative images are presented throughout. Fibers were not detected in the 0 mg/m^3^ lungs processed by either preparation method. Isolated mineral fibers from ashed lungs contained pitting and gaps in the fiber continuity and optically appeared to have been degraded and decomposed (Fig. 1A and D). Mineral fibers isolated from chemically digested lung lobes (Fig. 2) were not degraded and were similar in dimensions and aspect ratio of pre- and post-aerosolized/pre-inhaled LA 2007 [24, 25] . The mineral fibers isolated by chemical digestion produced SAED images and EDS spectra (Fig. 3) consistent with standards of amphibole asbestos from the Libby, MT region. EDS analysis further confirmed that the degraded/decomposed fibers were classified as non-asbestos mineral (NAM) with large amounts of silicon present (Fig. 3). Of note, a few non-degraded fibers were counted among the ashed samples and classified as LA fibers (Fig. 1E and F). SAED analysis of the ashed lung NAM fibers were featureless with no evidence of crystalline structure typical of Libby amphibole [15]. The results suggested chemical changes in the fibers as a result of ashing conditions were significant enough to decompose the mineral fibers and alter crystallinity.

The chemical composition of fibers collected from the lung lobe samples compared to pre-aerosolized LA 2007 is presented in Table 2. The oxide composition for the NAM fibers isolated from ashed lung lobes had higher Si and less Ca, Mg, and Fe than the fibers from chemically digested lung lobes. In addition, P was present in several NAM fibers (not typical in the composition of LA). From the ashed lobes, four types of fibers were identified by EDS analysis which included:

- NAM containing only Si,
- NAM containing Na, Si, P, and K,
- Amphibole asbestos consistent with that originating from the Libby, MT region (LA), and.
- A combination of Si and LA on the same fiber.

**Table 2:** Elemental oxide content of fibers collected from exposed lung samples

Study	Exposure Concentration (mg/m^3^)	Sample ID	Lobe	Fiber ID	Number Spectra Counted	Elemental Oxide Content (Weight%)^a^							
						CaO	MgO	SiO_2_	FeO	K_2_O	Na_2_O	Al_2_O_3_	P_2_O_5_
1 (with agarose gel)	NA	Bulk^b^	NA	LA	80	11.9 ± 1.6	17.9 ± 1.2	57.5 ± 1.3	10.1 ± 1.8	0.9 ± 0.5	2.0 ± 0.6	ND	ND
	0.1	Ashing	Left	LA	1	6.8	15.1	69.3	6.7	0.2	2.0	ND	ND
			Right		2	6.3	12.7	69.8	5.0	0.0	1.4	0.0	9.7
			Left	NAM	1	0.6	1.9	93.1	2.4	0.4	1.7	ND	ND
			Right		2	0.5	1.5	87.4	1.4	1.6	0.4	0.5	8.4
		Chemical Digestion	Left	LA	2	8.7	21.7	56.9	6.7	0.5	2.7	ND	5.5
			Right		1	8.4	22.5	61.4	4.8	0.7	2.1	ND	ND
	10	Ashing	Left	LA	2	3.0	6.2	81.5	6.7	0.1	2.5	ND	ND
			Right		1	8.1	15.3	66.8	6.8	1.0	2.1	ND	ND
			Left	NAM	2	0.6	1.9	94.9	ND	1.2	ND	ND	9.1
			Right		1	ND	ND	ND	ND	ND	ND	ND	ND
		Chemical Digestion	Left	LA	1	8.5	22.0	56.0	8.0	0.0	5.6	ND	ND
			Right		2	4.2	18.8	68.9	6.3	0.2	1.6	ND	ND
2 (without agarose gel)	NA	Bulk^c^	NA	LA	900	9.1 ± 1.4	21.6 ± 1.3	59.6 ± 2.2	5.4 ± 1.1	0.8 ± 0.3	3.5 ± 0.8	ND	ND
	10	Ashing	Both	LA	80	5.9 ± 3.8	15.9 ± 6.5	72.3 ± 12.5	2.9 ± 1.8	0.9 ± 0.3	2.0 ± 2.2	1.0 ± 1.4	ND
				NAM	80	1.3 ± 2.0	3.5 ± 4.6	85.1 ± 13.4	1.6 ± 1.5	2.7 ± 1.3	3.4 ± 2.3	20.3 ± 28.7	7.6 ± 8.5
		Chemical Digestion	Both	LA	80	7.2 ± 1.7	21.1 ± 4.1	63.8 ± 7.0	5.0 ± 1.5	0.6 ± 0.5	2.3 ± 2.1	ND	ND

^a^Data shown are average or/± standard deviation

^b^Wang et al, 24 Part A

^c^Wang et al, 25 Part B

*NA* Not applicable, *ND* Not detected, *LA* Libby amphibole asbestos, *NAM* Non-asbestos mineral

A summary of mineral fiber dimensions, particle size distribution, and determined fiber burden from the lung lobe samples compared to pre-aerosolized (bulk) material is presented in Table 3. The impact of the lobe origins (left vs. right) was also assessed; however, lobe origin did not show any differences in dimensions or chemical composition for either method. Therefore, the data are shown as combined results. As the primary purpose of this work was to determine the most effective preparation method for sample analysis for pulmonary tissues to determine fiber burden, the number of animals exposed was limited and thus, only large-scale differences would likely be noticeable between the lobes. In addition, sex or species differences (Wistar Han vs. Sprague Dawley) were not noted between the two sets of exposures and results.

**Fig. 4:**
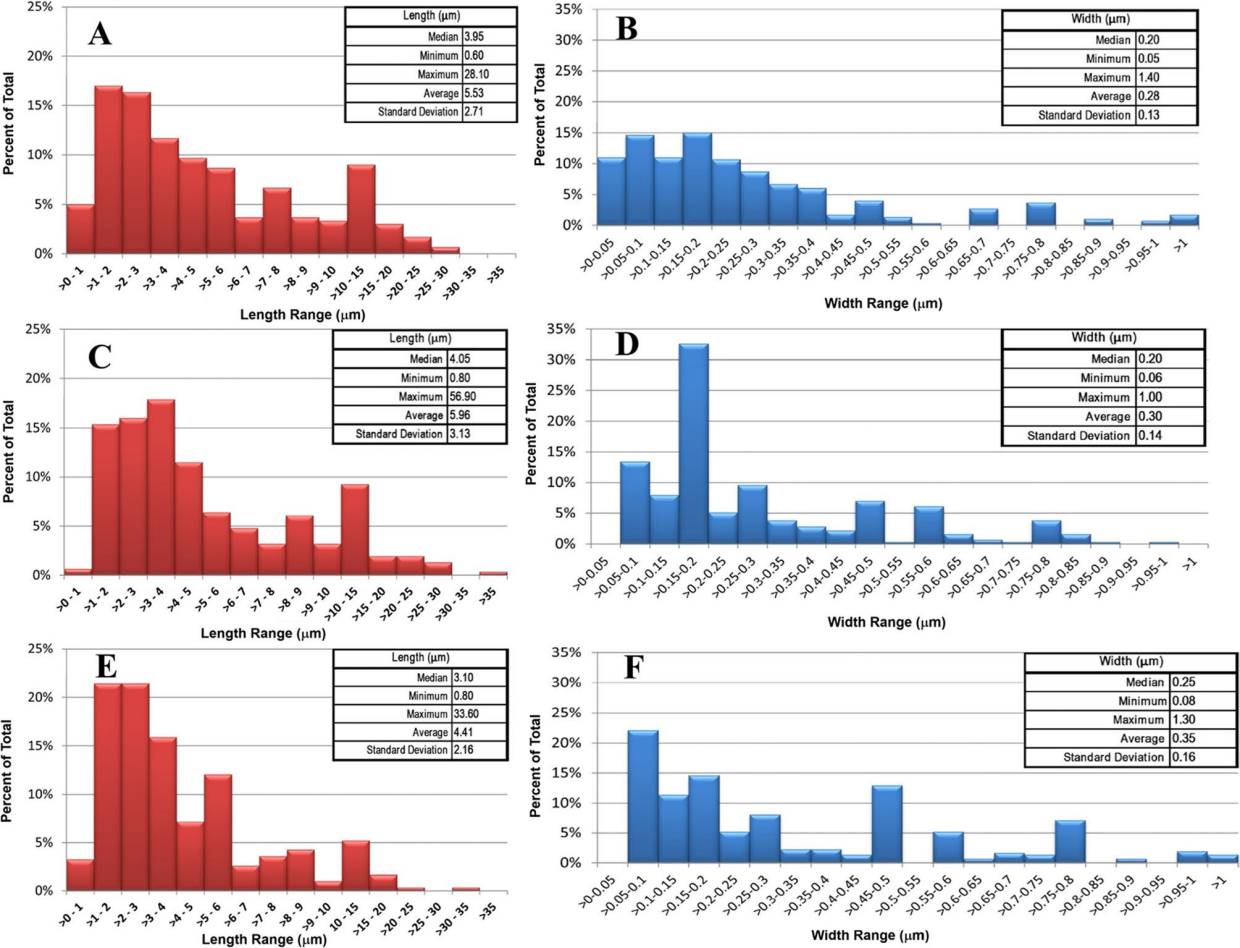
Length and Width Histograms: (**A**-**B**) Pre-Aerosolized LA 2007, (**C**-**D**) Ashed Lung NAM Fibers, (**E**-**F**) Digested Lung LA Fibers

Representative size distribution plots for the ashed and chemically digested fibers are shown in Fig. 4. Since the majority of the fibers present in the ashed lung lobes were NAM fibers with very few LA fibers observed, a LA size distribution plot was not generated. The fibers from ashed lung lobes (NAM + LA) were similar in length but slightly wider than fibers of the of pre- and post-aerosolized/pre-inhaled LA 2007. The analysis of bulk LA 2007 material did not show the presence of NAM fibers. Assuming the NAM fibers detected in the ashed lung tissues were LA fibers altered to transitional fibers while undergoing ashing in the furnace, a comparison of appropriate total fiber (NAM + LA) burden was performed. While the sizes of the fibers were similar between the ashed and chemically digested lung lobes, the burden data indicated chemical digestion resulted in better recovery of LA fibers as compared to the results of the ashed samples where the NAM fibers were a larger contributor to the fiber burden. The results indicated the ashing method (which is traditionally used in the isolation of asbestos fibers from building materials) is not suitable for studying the mineralogy of asbestos-containing lung samples.

**Table 3:** Particle size distribution and Libby amphibole asbestos lung lobe burden determination

Sample Preparation	Properties		Lung Lobe Samples (Exposure Concentration, mg/m^3^/Lobe)						
			**Study 1 (with agarose gel)**					**Study 2 (without agarose gel)**	
			**Bulk** ^**a, b**^	**0.1**		**10**		**Bulk** ^**a, c**^	**10**
				**Left**	**Right**	**Left**	**Right**		**Combined** ^**d**^
Ashing	Number of Samples		4	1	2	2	1	3	4
	Total Number Fibers Counted		424	34	28	76	72	900	1215
	Length (µm)	Average^e^	5.76 ± 2.55	4.61 ± 0.67	5.02 ± 1.05	4.49 ± 1.31	5.44 ± 1.45	4.71 ± 2.45	4.96 ± 4.16
		Range	0.25–28.30	1.85–12.73	1.41–20.82	0.71–22.00	0.99–32.76	0.50–33.60	0.53–39.20
	Width (µm)	Average^e^	0.33 ± 0.04	0.38 ± 0.05	0.44 ± 0.08	0.46 ± 0.14	0.45 ± 0.09	0.27 ± 0.14	0.50 ± 0.46
		Range	0.06–2.00	0.12–0.94	0.05–1.39	0.06–1.88	0.06–1.17	0.05–4.70	0.06–6.15
	Average Aspect Ratio^f^		17	12	12	10	12	23 ± 25	12 ± 9
	Average Percent PCMe Fibers		35	4	6	20	19	26	34
	Average LA Burden^g^ (1 × 10^6^ structures/g lung)		NA	0.237	0.0987	2.01	1.14	NA	< 0.166
	Average NAM Burden^g^ (1 × 10^6^ structures/g lung)		NA	0.434	0.485	0.913	0.333	NA	7.40 ± 4.37
	Percent LA in Total Structures		NA	35	18	81	77	NA	1
Chemical Digestion	Number of Samples		4	2	1	1	2	3	4
	Total Number Fibers Counted		424	72	16	57	57	900	1228
	Length (µm)	Average^e^	5.76 ± 2.55	5.99 ± 1.81	8.81 ± 1.26	3.87 ± 0.81	4.38 ± 1.23	4.71 ± 2.45	4.90 ± 4.28
		Range	0.25–28.30	0.94–37.04	0.57–48.62	0.71–15.44	0.51–15.91	0.50–33.60	0.52–56.90
	Width (µm)	Average^e^	0.33 ± 0.04	0.35 ± 0.15	0.26 ± 0.03	0.30 ± 0.06	0.34 ± 0.08	0.27 ± 0.14	0.33 ± 0.22
		Range	0.06–2.00	0.05–5.79	0.07–0.59	0.04–0.91	0.04–1.36	0.05–4.70	0.60–1.90
	Average Aspect Ratio^f^		17	17	34	13	13	23 ± 25	17 ± 14
	Average Percent PCMe Fibers		35	27	8	13	15	26	32
	Average LA Burden^g^ (1 × 10^6^ structures/g lung)		NA	1.43	0.316	9.40	7.04	NA	10.6 ± 9.2
	Average NAM Burden^g^ (1 × 10^6^ structures/g lung)		NA	< 0.0596	< 0.0590	< 0.521	< 0.472	NA	< 0.124
	Percent LA in Total Structures		NA	100	100	100	99	NA	100

^a^Bulk samples were not processed using either high temperature ashing or chemical digestion

^b^Wang et al., 24 Part A

^c^Wang et al, 25 Part B, Supplemental material

^d^There were no obvious differences in results for left vs. right lobe or male vs female, therefore results were combined under each preparation method

^e^Data shown are average ± standard deviation, based on numbers of fibers counted

^f^AR calculated from average length and width values for Study 1; AR calculated from the population results in Study 2

^g^For Study 1, n < 3, so no standard deviation was determined; for Study 2, n = 4

*NA* Not applicable, *LA* Libby amphibole asbestos, *NAM* Non-asbestos mineral, *PCMe* Phase Contrast Microscopy equivalent

## Discussion

Quantifying the number of inhaled mineral fibers and understanding their chemical and physical properties is critical to understanding and characterizing the hazards associated with these materials. Doing so requires proper fiber isolation from biological matrices followed by evaluation of physical size (length, width, aspect ratio), chemical composition, and quantitation of mineral constituents of fibers collected from the lungs. For meaningful interpretation, the methods for fiber isolation from pulmonary tissues should preserve the physical and chemical properties of the fibers as much as possible, such that they can be compared to those fibers in the exposure atmosphere and bulk material. Current methods used for isolation of inhaled mineral fibers include high temperature ashing or chemical digestion for isolation of inhaled mineral fibers in pulmonary tissue. To date, direct comparisons of the physical and chemical quality of inhaled mineral fibers when isolated by these methods described have not been reported.

The work presented herein aimed to identify the most appropriate method for sample preparation to assess inhaled mineral fiber burden. Preparation techniques for lung samples (both with and without agarose gel present), which were carefully examined in the present study, included high temperature ashing and chemical digestion. The differences in fiber morphology and chemical composition encountered in fibers following sample preparation from the lung samples for each preparation technique was evaluated.

Fiber degradation was first observed during the ashing process of exposed lungs infused with agarose gel. Under initial consideration as to the probable cause of the degradation was the ignition point of the agarose gel in the lung samples, which could have occurred during the high temperature ashing process resulting in microenvironments that would produce sufficient thermal energy to alter fibers. According to Ward et al. [23], LA changes to pyroxene at approximately 800–1000 °C. Kusiorowski et al. [12] reported that during thermal decomposition, tremolite (an amphibole in the LA suite of minerals) when fired at 1100°C, converted to diopside (pyroxene version of tremolite), enstatite (pyroxene version of anthophyllite) and cristobalite (SiO_2_). Subsequent benchtop analysis performed with LA 2007 spiked into agarose without lung material present did not recreate the altered fiber profile observed when agarose infused lung tissue was subjected to high temperature ashing (data not shown). This observation led to the consideration of a flash point occurring within the microenvironment of the lung alveolus, which in the presence of agarose, created isolated micro flashes of thermal energy of sufficient temperature and pressure to cause thermal degradation. Evaluation of this observation using lung tissue exposed to inhaled fibers but without agarose instilled into the lungs was then performed in the second study. Fiber degradation was again observed after ashing in the absence of agarose. The result of the second set of exposures indicated the presence of agarose did not induce the decomposition, rather something native to the lung tissue contributed to the degradation of the mineral fibers.

Ruling out the agarose as a cause for the high temperature decomposition opened the possibility to consider other sources, such as natural salts found in the body and lungs of rats. For example, calcium salts are effective in decreasing the decomposition temperatures of asbestos where the salt melt acts as a flux [5]. Lung damage via asbestos inhalation can be accompanied by dystrophic calcification in lung tissue [1]. For example, endoplasmic reticulum stress in lung tissue was shown to cause disruption in calcium homeostasis, resulting in increased amounts of calcium in macrophages associated with asbestos (e.g. chrysotile) damage [20]. In the context of the fiber results observed after ashing lung tissue in this paper, an increase in calcium in the vicinity of LA fibers in lung tissues would be consistent with the flux conditions noted by Fujishige [5]. Under an increased calcium environment the decomposition temperature of fibers would be decreased, allowing for decomposition to occur upon ashing. The end result would be fibers known to have been LA fibers upon exposure that do not have chemical compositions indicative of LA after isolation by ashing.

Chemical digestion of lung tissue, followed by filtration, resulted in consistent isolation of LA fibers that were unaltered. Fiber degradation was not observed when processing lung tissues using chemical digestion with or without agarose infusion. The chemical composition and fiber dimensions of fibers isolated by chemical digestion were consistent with the fibers characterized from the LA 2007 bulk material and from the exposure atmosphere [24, 25]. Optimization of sonication time and water temperature may be needed in subsequent studies to confirm full release of any fibers present on the PC filter.

The primary focus of the present study was to determine a suitable digestion method for pulmonary tissues analysis which can be applied to subsequent toxicological studies, including a more comprehensive study design with a larger number of animals. Thus, the critical step of the evaluation in this present work was to assess that a sufficient number of fibers could be retrieved from lungs/lobes without impact on fiber quality and chemistry assessment. The comparison of biological differences in study design is out of the scope of the present study. Moreover, the use of two different rat strains and sexes with similar results observed only adds confirmation to the theory that the high temperature decomposition was mostly likely due to the physical nature of the interior of the lungs in rats themselves.

The advantage of the chemical digestion method is that both size and composition of LA fibers from lung tissue can be consistently determined. In the high temperature ashing method, the size distribution analysis is consistent with bulk material analyses, although the mineralogical composition is not, as evidenced by the presence of NAM fibers.

## Conclusions

Fibers isolated from lung tissue by high temperature ashing were degraded, decomposed, and the resultant chemical profiles were not representative of LA 2007 used during the exposures. Fibers isolated from lung tissue by chemical digestion were similar in dimensions and aspect ratio to the bulk test material. Further, EDS analysis showed the chemical profile of the chemically digested fibers to be consistent with LA 2007. As the chemical digestion technique demonstrated acceptable recovery and consistent composition of LA 2007 fibers from exposed rat lungs, the chemical digestion method was deemed the preferred process for isolation of and toxicologic evaluation of inhaled mineral fibers. The use of agarose gel had no impact on the fibers and was not deemed necessary for the isolation of fibers in lung tissue.

Combined, the results of this study demonstrate the need for careful methodology considerations when isolating inhaled mineral fibers from biological matrices to ensure a consistent basis for sample preparation for future studies. To our knowledge, this study is the first comprehensive analysis of asbestos degradation in biological tissues and identifies an important aspect of compatibility to preparation techniques.

## Data Availability

Requests for the datasets used and analyzed during the current study should be made to the funding organization.

## Abbreviations

EDS: Energy Dispersive X-Ray Spectroscopy
EPA: Environmental Protection Agency
ISO: International Standards Organization
LA: Libby Amphibole Asbestos
LA 2007: Libby Amphibole Asbestos 2007
MCE: Mixed Cellulose Ester
NA: Not applicable
NAM: Non-Asbestos Mineral
ND: Not Detected
NIEHS: National Institute of Environmental Health Sciences
PC: Polycarbonate
PCMe: Phase Contrast Microscopy Equivalent
SAED: Selected Area Electron Diffraction
TEM: Transmission Electron Microscopy
